# Forensic psychiatric analysis of organic personality disorders after craniocerebral injury in Shanghai, China

**DOI:** 10.3389/fpsyt.2022.944888

**Published:** 2022-07-29

**Authors:** Beixu Li, Youxin Fang, Junyi Lin, Xueyan Chen, Chenhu Li, Meng He

**Affiliations:** ^1^School of Policing Studies, Shanghai University of Political Science and Law, Shanghai, China; ^2^Shanghai Fenglin Forensic Center, Shanghai, China; ^3^Department of Neurology, Minhang Hospital, Fudan University, Shanghai, China; ^4^Department of Forensic Medicine, School of Basic Medical Sciences, Fudan University, Shanghai, China; ^5^Shanghai Xuhui Mental Health Center, Shanghai, China

**Keywords:** forensic psychiatry, craniocerebral trauma, personality disorders, clinical manifestations, Neuroticism Extraversion Openness Five-Factor Inventory

## Abstract

**Objective:**

To explore the incidence rate and the differences of clinical manifestations of organic personality disorders with varying degrees of craniocerebral trauma.

**Materials and methods:**

According to the International Classification of Diseases-10, 1,027 subjects with craniocerebral trauma caused by traffic accidents were reviewed, the degrees of craniocerebral trauma were graded and those with personality disorder after craniocerebral trauma were diagnosed. The personality characteristics of all patients were evaluated by using the simplified Neuroticism Extraversion Openness Five-Factor Inventory (NEO-FFI).

**Results:**

The incidence rate of organic personality disorder after all kinds of craniocerebral trauma was 33.1%, while it was 38.7 and 44.2% in the patients after moderate and severe craniocerebral trauma, respectively, which was significantly higher than that in the patients after mild craniocerebral trauma (18.0%) (*P* < 0.05). Compared with the patients without personality disorder, the neuroticism, extraversion and agreeableness scores all showed significantly differences (*P* < 0.05) in the patients with personality disorder after craniocerebral trauma; especially the conscientiousness scores showed significant differences (*P* < 0.05) in the patients with personality disorder after moderate and severe craniocerebral trauma. The agreeableness and conscientiousness scores in the patients with personality disorder after moderate and severe craniocerebral trauma were significantly lower than that after mild craniocerebral trauma, and the patients with personality disorder after severe craniocerebral trauma had lower scores in extraversion than that after mild craniocerebral trauma.

**Conclusion:**

The severity and area of craniocerebral trauma is closely related to the incidence rate of organic personality disorder, and it also affects the clinical manifestations of the latter, which provides a certain significance and help for forensic psychiatric appraisal.

## Introduction

The estimated prevalence rate of any personality disorder in the general population was 3.9–15.7% with a worldwide pooled median prevalence at 7.8%, and the prevalence in China is considered below the median (1–7). Personality disorder is one of the main manifestations of mental disorders caused by traumatic brain injury (TBI), which seriously affects the family life, work and social functions of the injured. According to relevant literatures (8–10), the incidence rate of personality disorders after TBI is about 33.3%, and it goes to 59.1% when the TBI is severe. Moreover, some Chinese literatures and others reported the incidence rate might range from 10 to 92.8% (11, 12). However, it is disputable whether the incidence rate and clinical manifestations of personality disorders caused by traumatic brain injury are relevant to the craniocerebral injury severity. There were some Chinese researches focusing on the issues (13, 14), but a consensus has not been reached. A larger clinical data samples reaching to 1,027 patients from 2013 to 2018 were analyzed in this retrospective study in order to explore the incidence rate and the differences of clinical manifestations of organic personality disorders with varying degrees of craniocerebral trauma.

## Materials and methods

### Study design

This study was a retrospective study based on records of forensic appraisal cases of craniocerebral injury caused by traffic accidents at the Forensic Identification Center of Shanghai Medical College of Fudan University over a 5-year period from September 2013 to August 2018. Cases were accepted in this study according to the following criteria: (1) Head injury caused by traffic accident; (2) Age between 18 and 60 years old; (3) Complete data were available; (4) End of treatment period, disease period more than 6 months; (5) Forensic appraisal was conducted by forensic medical examiners. And cases were eliminated by the exclusive criteria: (1) Those with a history of mental disorders or substance abuse; (2) Those with a history of brain disease or previous traumatic brain injury; (3) Those with intellectual disability before injury; (4) Those with psychiatric symptoms attack during the forensic appraisal or non-cooperators. A total of 1,027 cases met the study criteria. Two reviewers independently extracted data from each of the cases. Disagreements between reviewers were resolved through discussions and a final consensus. Their archives were kept in the Forensic Identification Center of Shanghai Medical College of Fudan University records office. This study was approved by the Fudan University Ethics Committee. Written informed consent was given by each wounder person's next of kin, respectively, who was told at the time of the forensic appraisal that the relevant information and data might be used in scientific research. All cases were anonymized and de-identified prior to analysis.

### Diagnosis and assessment

The enrolled data showed that two associate chief physicians of psychiatry had conducted psychiatric examination and evaluation on the patient, while recording the patient's past history, personal history, and post-injury manifestations, excluded disguise, and made a diagnosis according to the International Classification of Diseases-10, ICD-10 diagnostic criteria. The OPD patients were diagnosed before the study begins. If they had seen a psychiatrist after the injury, they were diagnosed before the forensic appraisal and the diagnosis were confirmed by two associate chief physicians of psychiatry during the forensic appraisal. If they had not seen a psychiatrist, they were diagnosed by two associate chief physicians of psychiatry during the forensic appraisal. Patients were assessed and scaled by a qualified psychometrician. The diagnostic criteria for ICD-10 Organic Personality Disorder are that, in addition to a history of brain disease, damage, or functional disorder or other evidence, two or more of the following characteristics are needed to make a clear diagnosis: (1) Adhering to goal-directedness persistent decline in the ability to perform activities, especially for activities that are time-consuming and delayed gratification; (2) Changes in emotional behavior, characterized by emotional instability, irritability, or short bursts of anger and aggressive behavior, in some cases apathy is more prominent; (3) Revealing needs and impulses without regard to consequences or social conventions; (4) Cognitive dysfunction; (5) Marked changes in the speed and flow of speech; (6) Marked changes in sexual behavior.

The personality characteristics of all patients were evaluated by using the simplified version of the Neuroticism Extraversion Openness Five-Factor Inventory (NEO-FFI) (15, 16), which is a five-point scale from 0 to 4, including five dimensions, Neuroticism (N), Extraversion (E), Openness (O), Agreeableness (A), and Conscientiousness (C).

According to the diagnostic classification of craniocerebral trauma in the first hospital, post-traumatic coma, and Glasgow Coma Scale (GCS) scores, grading standards of craniocerebral injury are divided into (1) mild traumatic brain injury, GCS 13–15 points; (2) Medium traumatic brain injury, GCS 9–12 points; (3) Severe traumatic brain injury, GCS ≤8 points.

### Statistical analysis

Statistical analysis of the data was performed by SPSS 11.5 software. The data were presented as the means-standard deviations (*SD*) for age, years of education, time interval between injury and forensic appraisal. The Student *t*-test was used to compare data in the different groups. According to the characteristics of data distribution, enumeration data were analyzed by χ^2^ test, and measurement data were analyzed by variance analysis and pairwise comparison. The difference was considered significant when *P* was <0.05 (17).

## Results

### General demographic characteristics

As shown in Table 1, among the 1,027 patients with craniocerebral injury, there were 561 males (54.6%) and 466 females (45.4%), the average age was (39.4 ± 12.1) years, and the average years of education was (10.1 ± 4.4) years. The average time interval between the brain injury and the forensic appraisal was (10.3 ± 3.1) months.

**Table 1:** General demographic characteristics with/without organic personality disorder.

	**Organic personality disorder (340)**	**No organic personality disorder (687)**	**The value of the statistics**	***P***
Gender			χ^2^ = 2.673	*P* = 0.102
Male	198 (58.2%)	363 (52.8%)		
Female	142 (41.8%)	324 (47.2%)		
Age	41.4 ± 13.3 years	37.9 ± 9.1 years	*t =* 1.476	*P* = 0.112
Years of education	8.8 ± 5.2 years	11.3 ± 3.8 years	*t =* 1.345	*P* = 0.143
Time interval between injury and forensic appraisal	9.3 ± 2.7 months	10.7 ± 3.4 months	*t =* 1.061	*P* = 0.203

Among them, 340 cases were diagnosed as organic personality disorder, accounting for 33.1% of the total number. There was no significant difference in gender (χ^2^ = 2.673, *P* > 0.05, Table 1). Besides the gender, the difference was not statistically significant in age (*t* = 1.476, *P* > 0.05, Table 1), years of education (*t* = 1.345, *P* > 0.05, Table 1), and evaluation time interval between the brain injury and the forensic appraisal (*t* = 1.061, *P* > 0.05, Table 1).

### The incidence rate of personality disorders after severe TBI was the highest

**Figure 1:**
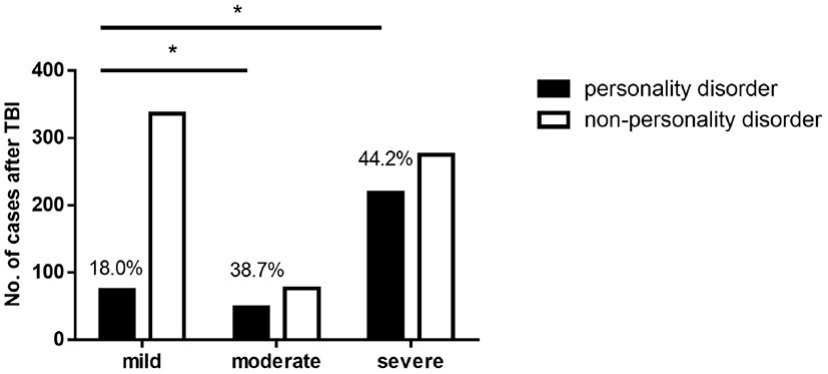
The incidence rate of personality disorders after TBI. *The difference was statistically significant compared with mild group. The incidence rates of personality disorders after mild, moderate and severe traumatic brain injury were 18.0, 38.7, and 44.2%. The incidence rate of personality disorder after moderate TBI was significantly higher than that after mild TBI (χ^2^ = 23.056, *P* < 0.05), and the incidence rate of personality disorder after severe TBI was also significantly higher than that after mild TBI (χ^2^ = 70.067, *P* < 0.05).

The results were displayed in Table 2 and Figure 1. Among the 1,027 cases of craniocerebral injury, 410 cases (39.9%) had mild injury, 124 cases (12.1%) moderate, and 493 cases (48.0%) severe. Among them, the incidence rates of personality disorders after mild, moderate, and severe traumatic brain injury were 18.0% (74 cases), 38.7% (48 cases), and 44.2% (218 cases), respectively, and the difference was statistically significant (χ^2^ = 71.225, *P* < 0.05). After pairwise comparison, it was found that the incidence rate of personality disorder after moderate was significantly higher than that after mild traumatic brain injury (χ^2^ = 23.056, *P* < 0.05), and the incidence rate of personality disorder after severe traumatic brain injury was also significantly higher than that after mild traumatic brain injury (χ^2^ = 70.067, *P* < 0.05). However, there was no significant difference in the incidence rate of personality disorders between after moderate traumatic brain injury and after severe traumatic brain injury (χ^2^ = 1.226, *P* > 0.05).

**Table 2:** The incidence rate of personality disorders after TBI.

	**Mild**	**Moderate**	**Severe**	**Total**
*N* (%)	410 (39.9%)	124 (12.1%)	493 (48.0%)	1,027 (100%)
Personality disorder	74	48	218	340
Incidence rate^£^	18.0%	38.7%^*^	44.2%^*^	33.1%

£ *The difference was statistically significant among mild, moderate and severe group*.

* *The difference was statistically significant compared with mild group*.

### The incidence rate of personality disorders after frontal and/or temporal lobe injury was higher

**Figure 2:**
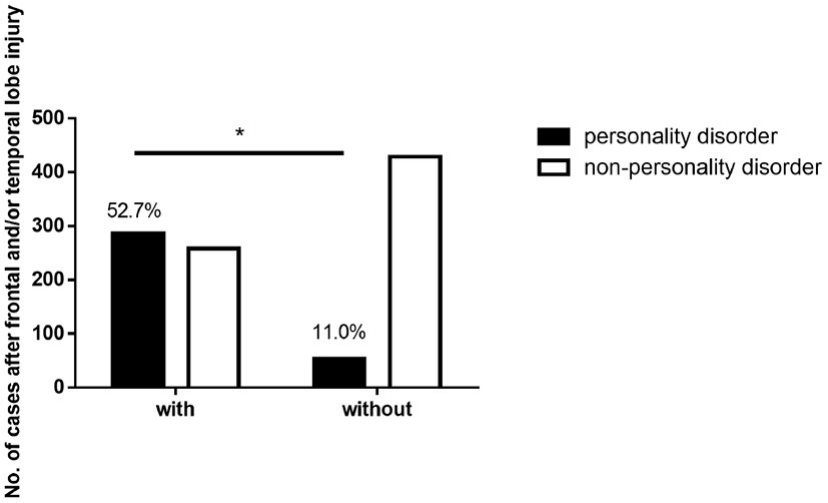
The incidence rate of personality disorders after frontal and/or temporal lobe injury. *The difference was statistically significant between two groups. The incidence rate of personality disorder after frontal and/or temporal lobe injury was 52.7% (287 in 545 cases). The incidence rate of personality disorder after neither frontal lobe nor temporal lobe damage was 11.0% (53 in 482 cases), which was significantly lower than the incidence rate after frontal and/or temporal lobe injury (χ^2^ = 200.5, *P* < 0.05).

Of the 1,027 cases, 545 (53.1%) had frontal and/or temporal lobe damage, and 482 (46.9%) had neither frontal lobe nor temporal lobe damage. As shown in Table 3 and Figure 2, the incidence rate of personality disorder after frontal and/or temporal lobe injury was 52.7% (287 in 545 cases). The incidence rate of personality disorder after neither frontal lobe nor temporal lobe damage was 11.0% (53 in 482 cases), which was significantly lower than the incidence rate after frontal and/or temporal lobe injury (χ^2^ = 200.5, *P* < 0.05).

**Table 3:** The incidence rate of personality disorders after frontal and/or temporal lobe injury.

	**Frontal and/or temporal lobe damage**		**Total**
	**Yes**	**No**	
*N* (%)	545 (53.1%)	482 (46.9%)	1,027 (100%)
Personality disorder	287	53	340
Incidence rate	52.7%	11.0%^*^	33.1%

* *The difference was statistically significant between two groups*.

### Clinical characteristics of personality disorders were correlated with different degrees of craniocerebral injury

**Figure 3:**
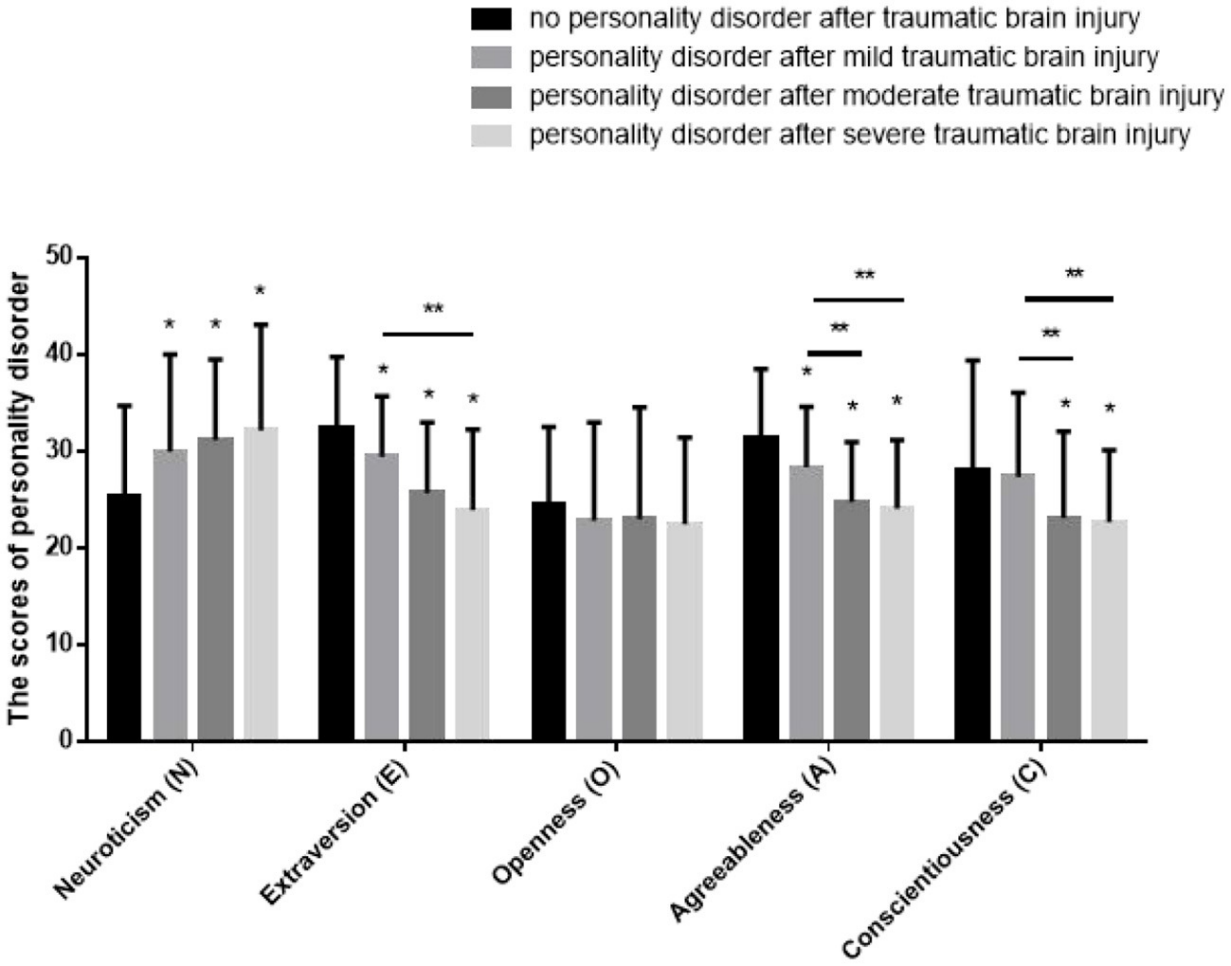
The scores in five dimensions of personality disorder. *Compared with people without personality disorder, *P* < 0.05. **Compared with people with personality disorder after mild traumatic brain injury, *P* < 0.05. The scores in neuroticism dimension of people with personality disorder after TBI were significantly higher than that of people without personality disorder. The scores in extraversion dimension of people with personality disorder after TBI were significantly lower than that of people without personality disorder. The scores in agreeableness dimension of people with personality disorder after TBI were significantly lower than that of people without personality disorder. The scores in conscientiousness dimension of people with personality disorder after moderate and severe TBI were significantly lower than that of people with personality disorder after mild TBI and without personality disorder.

The scores in five dimensions of people without personality disorder, people with personality disorder after mild traumatic brain injury, people with personality disorder after moderate traumatic brain injury and people with personality disorder after severe traumatic brain injury are shown in Table 4 and Figure 3.

**Table 4:** The scores in five dimensions of personality disorder.

	**Neuroticism (** ***N*** **)**	**Extraversion (E)**	**Openness (O)**	**Agreeableness (A)**	**Conscientiousness (C)**
No personality disorder after traumatic brain injury (*n* = 687)	25.37 ± 9.33	32.55 ± 7.21	24.59 ± 7.94	31.34 ± 7.19	28.10 ± 11.34
Personality disorder after mild traumatic brain injury (*n* = 74)	30.01 ± 10.03^*^	29.46 ± 6.24^*^	22.87 ± 10.13	28.37 ± 6.23^*^	27.38 ± 8.67
Personality disorder after moderate traumatic brain injury (*n* = 48)	31.17 ± 8.32^*^	25.75 ± 7.24^*^	23.01 ± 11.56	24.79 ± 6.16^*^, ^**^	23.14 ± 8.93^*^, ^**^
Personality disorder after severe traumatic brain injury (*n* = 218)	32.15 ± 10.96^*^	23.98 ± 8.31^*^, ^**^	22.49 ± 8.94	24.11 ± 7.08^*^, ^**^	22.71 ± 7.43*, ^**^

* *Compared with people without personality disorder, P < 0.05*.

** *Compared with people with personality disorder after mild traumatic brain injury, P < 0.05*.

The scores in neuroticism dimension of people with personality disorder after mild traumatic brain injury were significantly higher than that of people without personality disorder (*t* = 1.796, *P* < 0.05). The scores in neuroticism dimension of people with personality disorder after moderate traumatic brain injury were significantly higher than that of people without personality disorder (*t* = 1.833, *P* < 0.05). The scores in neuroticism dimension of people with personality disorder after severe traumatic brain injury were significantly higher than that of people without personality disorder (*t* = 2.015, *P* < 0.05). The difference between mild, moderate and severe traumatic brain injury was not statistically significant (*t* = 1.323, *P* > 0.05).

The scores in extraversion dimension of people with personality disorder after mild traumatic brain injury were significantly lower than that of people without personality disorder (*t* = 1.771, *P* < 0.05). The scores in extraversion dimension of people with personality disorder after moderate traumatic brain injury were significantly lower than that of people without personality disorder (*t* = 1.895, *P* < 0.05). The scores in extraversion dimension of people with personality disorder after severe traumatic brain injury were significantly lower than that of people without personality disorder (*t* = 1.943, *P* < 0.05), meanwhile they were significantly lower than that of people with personality disorder after mild traumatic brain injury as well (*t* = 1.753, *P* < 0.05).

There were no significant differences in the scores in openness dimension between people without personality disorder, people with personality disorder after mild traumatic brain injury, people with personality disorder after moderate traumatic brain injury and people with personality disorder after severe traumatic brain injury (*t* = 1.440, *P* > 0.05).

The scores in agreeableness dimension of people with personality disorder after mild traumatic brain injury were significantly lower than that of people without personality disorder (*t* = 1.761, *P* < 0.05). The scores in agreeableness dimension of people with personality disorder after moderate traumatic brain injury were significantly lower than that of people without personality disorder (*t* = 2.015, *P* < 0.05), meanwhile they were significantly lower than that of people with personality disorder after mild traumatic brain injury as well (*t* = 2.353, *P* < 0.05). The scores in agreeableness dimension of people with personality disorder after severe traumatic brain injury were significantly lower than that of people without personality disorder (*t* = 1.729, *P* < 0.05), meanwhile they were significantly lower than that of people with personality disorder after mild traumatic brain injury as well (*t* = 1.725, *P* < 0.05).

There were no significant differences in the scores in conscientiousness dimension between people without personality disorder and people with personality disorder after mild traumatic brain injury (*t* = 0.978, *P* > 0.05). The scores in conscientiousness dimension of people with personality disorder after moderate traumatic brain injury were significantly lower than that of people without personality disorder (*t* = 2.060, *P* < 0.05), meanwhile they were significantly lower than that of people with personality disorder after mild traumatic brain injury as well (*t* = 1.943, *P* < 0.05). The scores in conscientiousness dimension of people with personality disorder after severe traumatic brain injury were significantly lower than that of people without personality disorder (*t* = 2.074, *P* < 0.05), meanwhile they were significantly lower than that of people with personality disorder after mild traumatic brain injury as well (*t* = 2.015, *P* < 0.05).

## Discussion

In the process of forensic psychiatric appraisal, there are many cases of disguised personality disorder due to various motives, and the diagnosis of personality disorder is still largely dependent on the medical history materials and the subjective performance of the injured. Therefore, how to identify the authenticity of post-traumatic personality disorder is very important for forensic psychiatric appraisal. This study in which over 1,000 cases were reviewed shows that, compared with those without personality disorder after traumatic brain injury, individuals with personality disorder have higher level of neuroticism, lower extraversion, and lower agreeableness. People suffer from personality disorder after moderate or severe traumatic brain injury can also show low level of consciousness. Therefore, if the authenticity of personality disorder is suspected in the process of forensic psychiatric appraisal, the comprehensive analysis of the performance of the injured in different dimensions based on this study can be referred to, which is helpful to distinguish the disguise and improve the reliability of the forensic appraisal opinion.

The clinical manifestations of organic personality disorder caused by craniocerebral trauma are diverse (18, 19), and it is difficult to make a clear subtype classification according to the clinically applied diagnostic criteria. In the vast majority of cases, only a description of personality tendencies could be made. NEO-FFI is one of the most widely used personality assessment scales, and has been widely used in clinical psychology, psychopathology, industrial and management psychology, and other fields. In 1981, Goldberg proposed the “five-factor model” of personality on the basis of his predecessors. In 1985, the NEO-FFI personality questionnaire has been compiled, being introduced to China in 1996. The study of people suffering from psychiatric disorder shows the good applicability of NEO-FFI in China (20, 21).

Therefore, NEO-FFI was chosen in this study to reflect the personality tendencies of people with organic personality disorders caused by traumatic brain injury. The test results showed that, compared with those without personality disorder, the patients with personality disorder after mild traumatic brain injury had higher scores of neuroticism and lower scores of extraversion and agreeableness, and the differences were statistically significant (*P* < 0.05). In addition to higher scores of neuroticism and lower scores of both extraversion and agreeableness, the patients with personality disorder after moderate and severe traumatic brain injury also had lower scores of conscientiousness, and the differences were statistically significant as well (*P* < 0.05). Meanwhile, different degrees of craniocerebral injury show different characteristics in personality tendencies (22–24). Compared with post mild traumatic personality disorder, post moderate traumatic personality disorder showed lower level of agreeableness (rude action, suspicion, revenge, cruelty, etc.) and lower level of conscientiousness (weak will, laziness, rash behavior, etc.). Compared with post mild traumatic personality disorder, post severe traumatic personality disorder also showed lower level of extraversion (unconcern, few words, flinch, etc.) besides lower level of agreeableness and conscientiousness.

In terms of incidence rate, the conclusions were inconsistent due to factors such as diagnostic criteria, sample size, and differences in the time interval between studies and trauma. The incidence rate of personality disorder in China is considered below 7.8% (7, 25). Some Chinese literatures report that the incidence rate of personality disorder after traumatic brain injury is as high as 92.8%, while 10% is also reported. There are few studies in China on the influence of the degree of traumatic brain injury on personality disorders. The results of this study showed that the incidence rate after traumatic brain injury was 33.1%, much higher than 7.8%. This study revealed that the incidence rate of personality disorder correlated with traumatic brain injury.

A retrospective study on 177 adolescent patients with traumatic brain injury was conducted, which revealed that the severity of traumatic brain injury was an influencing factor for the occurrence of personality disorders (22). In this study, the incidence rate of personality disorder after mild traumatic brain injury was 18.0%, and the incidence rate of personality disorder after moderate and severe traumatic brain injury was 38.7 and 44.2%, respectively. The present results are relatively close to previous study result which showed the incidence rate of personality disorder after TBI was from 33.3 to 59.1%. This study confirms that the more severe the traumatic brain injury, the greater the possibility of suffering from personality disorders.

Previous studies have been carried out on organic personality disorders caused by craniocerebral trauma, mainly focusing on the incidence rate of personality disorders and relevant influencing factors (26–28). It is generally recognized that frontal and temporal lobe injuries are related to personality disorders (29, 30). The present study also showed that the incidence rate of personality disorders after frontal and/or temporal lobe damage was significantly higher than that without frontal and/or temporal lobe damage. The previous point of view is verified.

In conclusion, the severity of craniocerebral injury and the area of brain damage is related to the occurrence of personality disorders.

The specific mechanism of personality disorder after craniocerebral trauma still remains unclear now. In the past studies, personality disorders after traumatic brain injury might be possibly caused by the pattern of brain network damage (30), or traumatic brain injury might be just an inducing factor (31, 32). Combined with the results of colleagues and this study, it is believed that organic personality disorder can be directly caused by traumatic brain injury, because the different brain damage area brought the different incidence rate. The mechanism of causing organic personality disorder after traumatic brain injury remains to be further studied through experiment research. Since our study was a retrospective investigation which could not evaluate severity of personality disorder changing with time, prospective study is needed to conducted in the future to investigate it.

## Data availability statement

The raw data supporting the conclusions of this article will be made available by the authors, without undue reservation.

## Ethics statement

These studies involving humans was approved by the Fudan University Ethics Committee. Written informed consent to participate was given by each wounder person's next of kin respectively.

## Author contributions

BL and YF reviewed archives, extracted data, and wrote this manuscript. JL analyzed the data. XC, CL, and MH confirmed the diagnosis and provided advisory support of psychiatry. MH conceived and designed this study. All authors contributed to the article and approved the submitted version.

## Funding

This study is supported by Shanghai Key Laboratory of Crime Scene Evidence, Institute of Forensic Science (2020XCWZK01). The funder had no role in study design, data collection and analysis, decision to publish, or preparation of the manuscript.

## Conflict of interest

The authors declare that the research was conducted in the absence of any commercial or financial relationships that could be construed as a potential conflict of interest.

## Publisher's note

All claims expressed in this article are solely those of the authors and do not necessarily represent those of their affiliated organizations, or those of the publisher, the editors and the reviewers. Any product that may be evaluated in this article, or claim that may be made by its manufacturer, is not guaranteed or endorsed by the publisher.
